# Detection of African swine fever virus genotype XV in a sylvatic cycle in Saadani National Park, Tanzania

**DOI:** 10.1111/tbed.13747

**Published:** 2020-08-15

**Authors:** Emma Peter, Eunice Machuka, Dedan Githae, Edward Okoth, Sarah Cleaveland, Gabriel Shirima, Lughano Kusiluka, Roger Pelle

**Affiliations:** ^1^ Biosciences eastern and central Africa – International Livestock Research Institute Hub Nairobi Kenya; ^2^ Nelson Mandela African Institution of Science and Technology Arusha Tanzania; ^3^ Sokoine University of Agriculture Morogoro Tanzania; ^4^ Institute of Biodiversity, Animal Health and Comparative Medicine College of Medical, Veterinary and Life Sciences University of Glasgow Glasgow UK

**Keywords:** ASF, ASFV, ELISA, *Ornithodoros*, PCR, warthogs

## Abstract

African swine fever (ASF) is a severe haemorrhagic disease of domestic pigs caused by ASF virus (ASFV). ASFV is transmitted by soft ticks (*Ornithodoros moubata* complex group) and by direct transmission. In Africa, ASF is maintained in transmission cycles of asymptomatic infection involving wild suids, mainly warthogs (*Phacochoerus africanus*). ASF outbreaks have been reported in many parts of Tanzania; however, active surveillance has been limited to pig farms in a few geographical locations. There is an information gap on whether and where the sylvatic cycle may occur independently of domestic pigs. To explore the existence of a sylvatic cycle in Saadani National Park in Tanzania, blood and serum samples were collected from 19 warthogs selected using convenience sampling along vehicle‐accessible transects within the national park. The ticks were sampled from warthog burrows. Blood samples and ticks were subjected to ASFV molecular diagnosis (PCR) and genotyping, and warthog sera were subjected to serological (indirect ELISA) testing for ASFV antibody detection. All warthog blood samples were PCR‐negative, but 16/19 (84%) of the warthog sera were seropositive by ELISA confirming exposure of warthogs to ASFV. Of the ticks sampled, 20/111 (18%) were positive for ASFV by conventional PCR. Sequencing of the p72 virus gene fragments showed that ASF viruses detected in ticks belonged to genotype XV. The results confirm the existence of a sylvatic cycle of ASFV in Saadani National Park, Tanzania, that involves ticks and warthogs independent of domestic pigs. Our findings suggest that genotype XV previously reported in 2008 in Tanzania is likely to be widely distributed and involved in both wild and domestic infection cycles. Whole‐genome sequencing and analysis of the ASFV genotype XV circulating in Tanzania is recommended to determine the phylogeny of the viruses.

## 1 INTRODUCTION

African swine fever is a severe haemorrhagic fever that affects swine species. The disease is notifiable to the World Organisation for Animal Health and is listed as the highest priority disease of domestic pigs in many affected countries. There is currently no vaccine for the control of ASF (Arias et al., 2017). The approaches available for control include early detection of cases followed by stamping out and enforcement of strict biosecurity measures to prevent further spread (Penrith, 2009; Woźniakowski, Frączyk, Niemczuk, & Pejsak, 2016). The disease affects wild boars and naive domestic pigs, causing high mortality. The disease is associated with high economic losses and reduction of animal‐source protein for many poor households, thus contributing to food insecurity (Cappai, Rolesu, Coccollone, Laddomada, & Loi, 2018).

The causative agent for ASF is a double‐stranded DNA virus of the family *Asfarviridae*. It is the only member of the group and the only known arthropod‐borne DNA virus capable of causing haemorrhagic fever. The virus genome size ranges from 175 to 190 kbp depending on the genotype. To date, 24 genotypes of the virus have been identified based on the p72 genetic region of the virus (Quembo, Jori, Vosloo, & Heath, 2018). Of the known ASFV genotypes, a broad ASFV diversity has been found in the South‐eastern part of Africa (Boshoff, Bastos, Gerber, & Vosloo, 2007; Lubisi, Bastos, Dwarka, & Vosloo, 2005, 2007). Three cycles are known to be responsible for the spread and maintenance of ASFV in Africa. A sylvatic cycle between a tick vector belonging to the *Ornithodoros moubata* complex (usually *Ornithodoros porcinus porcinus*) and warthogs (*Phacochoerus africanus*) has been characterized in the past (Abworo, 2012; Anderson, Hutchings, Mukarati, & Wilkinson, 1998; Bastos, Arnot, Jacquier, & Maree, 2009; Lubisi et al., 2005; Plowright, Parker, & Peirce, 1969; Ravaomanana et al., 2011). Bush pigs (*Potamochoerus larvatus*) and giant forest hogs (*Hylochoerus meinertzhageni*) can be infected, but their role in maintenance and transmission of the disease, if any, remains unknown. The second cycle involves the tick vector and domestic pigs, and the third, considered as the most important, is the pig‐to‐pig cycle through direct contact, also known as the domestic cycle. The fourth ASFV transmission cycle, typical in Europe and Asia, is associated with the wild boars (*Sus scrofa*).

Several studies have been carried out in South‐eastern Africa to identify the possible mechanisms for ASF maintenance between outbreaks (Abworo et al., 2017; Gallardo et al., 2011; Lubisi et al., 2005, 2007; Penrith, 2013). The sylvatic cycle and carrier pigs have both been suggested to be associated with the virus persistence in East Africa (Abworo, 2012). However, most occurrences in the region have not been directly linked to ticks and or wild hosts as the source (Sanchez‐Vizcaino, Mur, Bastos, & Penrith, 2015). A previous study done in 1968 at Kirawira area within the Serengeti National Park in Tanzania showed evidence of a 15% infection prevalence of *Ornithodoros* ticks collected from warthog burrows (Plowright et al., 1969). There is also a body of evidence in the literature on the tick's ability to maintain and spread the virus through venereal, transovarial and transstadial transmissions (Review from Kleiboeker & Scoles, 2001). Once the disease has been established in pigs, numerous potential routes of pig‐to‐pig transmission arise, for example through feeding on contaminated products (swill and garbage) and contact with contaminated fomites. The free‐ranging movements and scavenging behaviour of many domestic pigs in Africa, inadequate levels of biosecurity plus the illegal and uncontrolled movement of pigs are among the risk factors contributing to the spread of the virus (Wozniakowski *et al*., 2016; Penrith, 2009). The importance of the sylvatic cycle and its hosts in Sub‐Saharan Africa and the Indian ocean region has been discussed in detail in a previous review (Jori et al., 2013).

Although ASF has been reported in various parts of Tanzania including but not limited to Kilimanjaro, Arusha, Mbeya, Iringa, Morogoro, Pwani and Dar es salaam regions, surveillance aimed at identifying circulating genotypes has been limited to outbreaks from pig farms in a few geographical locations (Chang'a et al., 2019; Misinzo et al., 2011, 2014). A study conducted in Serengeti National Park in Tanzania upon warthogs revealed ASFV seropositivity in all sampled warthogs, but the genetic characterization of the associated virus was not done (Katale, Fyumangwa, Mdaki, & Hoare, 2012). Data are still needed in areas where the sylvatic cycle may occur independently of domestic pigs to investigate the relationship between ASF viruses circulating in different epidemiological cycles. This study, therefore, investigated the occurrence and characteristics of an ASFV sylvatic cycle in Saadani National Park, Tanzania, a protected ecosystem that has no neighbouring domestic pig populations.

## 2 MATERIALS AND METHODS

### 2.1 Description of the study area

**FIGURE 1:**
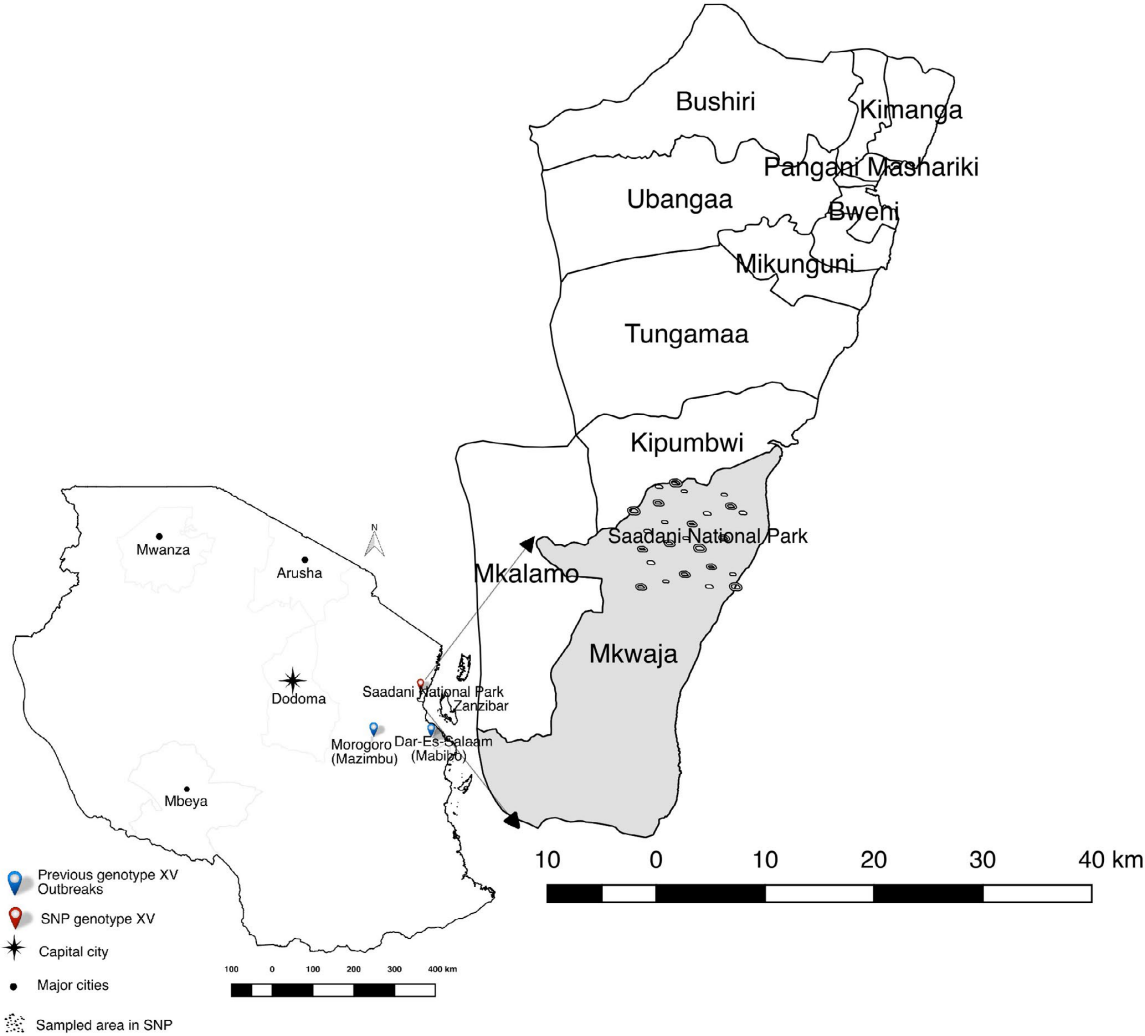
A map of Tanzania showing previous genotype XV outbreaks and sampled area in Saadani National Park

This study was conducted in Saadani National Park (SNP), located in Tanga district on the North‐eastern coast of Tanzania (Figure 1). The warthogs in the park closely interact with human dwellings and occasionally get inside the human residences. The park is unfenced and is located between two villages, Mkwaja and Saadani, where the major socio‐economic activities include fishing, tour guiding and salt mining which are carried out alongside conservation activities.

Saadani National Park covers an area of 1,062 km^2^ and is the only park with coastal‐based wildlife conservation activities in Tanzania. The ecosystem falls into three districts which are Handeni and Pangani districts in Tanga region and Bagamoyo district in Pwani Region. The distance from Saadani to the nearby major towns is 150 km from Bagamoyo, 200 km from Dar es Salaam, 75 km from Pangani and 40 km from Zanzibar. The park is composed of the former Mkwaja ranch, Zaraninge Forest Reserve and Saadani Game Reserve which was incorporated into the Tanzania National Parks in 2005. Saadani has a coastal climate that is hot and humid with an average temperature between 25 and 30ºCthroughout the year (Baldus, Roettcher, & Broska, 2001), with an annual rainfall of between 500 and 1,700 mm (and a mean of 900 mm). The vegetation cover in the park includes mangrove forests around the Wami River to the ocean with short and tall grass savannah and black cotton plains in most areas (Cochard & Edwards, 2003). Warthogs are very common in Saadani.

Saadani National Park has three main components identified as the former Mkwaja ranch, the Zaraninge Forest Reserve and the former Saadani Game Reserve (Figure 1). This study took place in the former Saadani Game Reserve which covers an area of around 200 km^2^ while excluding the two other components. Preliminary visits to communities surrounding the park determined that the ethnicity of communities surrounding the park is mainly Wazigua and Wadoe with the main religion being Islam. In Islam, the meat from pigs is considered unclean. Coming into contact with pigs is regarded as dirty and as a result, no pigs are kept in these communities. Having no interface with domestic pigs or pig farms, Saadani is a suitable site to study a pure sylvatic host ASFV life cycle. Domesticated animals observed in communities surrounding the park include sheep, goats, cattle and chickens.

### 2.2 Warthogs sampling

Three veterinarians handled warthogs of which two were wildlife veterinarians, and one was a wildlife research scientist. Two park guards ensured the safety of staff from wild animals during sampling and security of the animals after sample collection. Six days were dedicated to this activity. The first day was used for area survey and planning, three days for warthog sampling and two days for tick sampling.

Convenience sampling was carried out with darting locations selected based on the accessibility of the site. Immobilization of warthogs was carried out at cooler times of day between 6 to 10 a.m. and 4 to 6 p.m. when warthogs graze in open areas of the park and thus easy to locate. Warthogs were darted from a vehicle using a compressed dart gun with plastic darts (Dan inject^®^, DanWild LCC). The darts were loaded with a dosage of 0.04 mg/kg body weight of etorphine hydrochloride (M99^®^, Novartis, as an immobilizer at an approximate distance of fewer than 10 m. From the jugular vein, 10 ml of blood was collected using a 16‐gauge needle. The blood was quickly aliquoted into plain and EDTA‐treated vacutainer tubes. Sampled animals were sprayed with a coloured wound spray, oxytetracycline (Alamycin^®^, Norbrook Laboratories) at the needle injection site for both disinfection and subsequent identification to avoid repeat sampling. The immobilization effects were reversed by intramuscular injection of 0.04 mg/kg body weight diprenorphine hydrochloride (M5050^®^, Novartis).

Data on age, sex and location of the animal were collected. A total of 19 blood and 19 sera samples were collected for further laboratory analyses. Warthogs were sampled without replacement.

### 2.3 Collection of soft ticks from warthog burrows

Spotting all the burrows in the area could require walking in the bush, which was dangerous in the park containing predators such as lions. Based on such limitations and time constraint, 12 warthog burrows were identified of which only five were active. Burrows were located approximately one to four minutes walk apart, although it could take longer to reach for those in bushier areas. Active warthog burrows were identified following confirmation of the presence of warthogs in the burrows the night before sampling. Two days were dedicated for this activity during which all the 12 burrows were examined for the existence of ticks, although only the five active burrows had ticks. Each burrow was considered a sampling stratum in which every tick was a sample unit. For safety reasons, ticks were collected from the burrows when animals were out for feeding. The sand was scooped from deep down in the burrows using a long‐handled spade, spread on a black plastic sheet and exposed to strong sunlight for 3 min to stimulate movement and identification of ticks. The collected ticks were placed into falcon tubes with perforated tops covered with a cotton cloth material to ensure containment and ventilation. Once in the laboratory, ticks were preserved in 70% ethanol at +4ºC until analysis.

### 2.4 Handling of samples in the field

Blood was collected in both plain and EDTA‐treated 5ml vacutainer tubes. Immediately after collection, the blood in a plain tube was put in the rack for 6h to allow separation of the clot from serum. The serum was collected using disposable pipettes and transferred into Eppendorf tubes. The blood in EDTA tubes and sera in Eppendorf tubes were immediately put in a portable fridge at +4°C until they reached the laboratory for more extended storage and analysis.

### 2.5 Processing of samples and data

#### 2.5.1 ASFV antibody detection by ELISA

An indirect Enzyme‐Linked Immunosorbent Assay (i‐ELISA) was performed on warthog serum samples using the African Swine Fever Indirect‐Screening test (ID‐VET, Grabels, France) according to the manufacturer's instructions. The test was considered valid if the optical density (OD) of the positive control was more than 0.35, and the ratio between positive and negative control ODs was more than 3.0. Our test detected the OD of 1.66 in the positive control and a ratio of 3.91, thereby meeting the necessary criteria. Samples were considered positive if the sample to positive control percentage (S/P%) was at least 40% (used as a cut‐off point in Figure 3) (Cubillos et al., 2013).

#### 2.5.2 DNA extraction and PCR detection of ASFV in warthog blood

DNA from blood samples was purified using the DNeasy™ Blood and Tissue Kit (Qiagen, Hilden, Germany) as per the manufacturer's guidelines. A negative extraction control containing only PBS was included in place of a blood sample.

A 257bp region corresponding to the C‐terminal region of the p72 major capsid protein encoded by the B646L gene (*VP72*) was amplified using the diagnostic primers PPA1 and PPA2 as previously described by Agüero et al. (2003). PCR reactions were performed in a total volume of 25 μl following standard PCR techniques.

#### 2.5.3 DNA extraction and detection of ASFV in soft ticks

**FIGURE 2:**
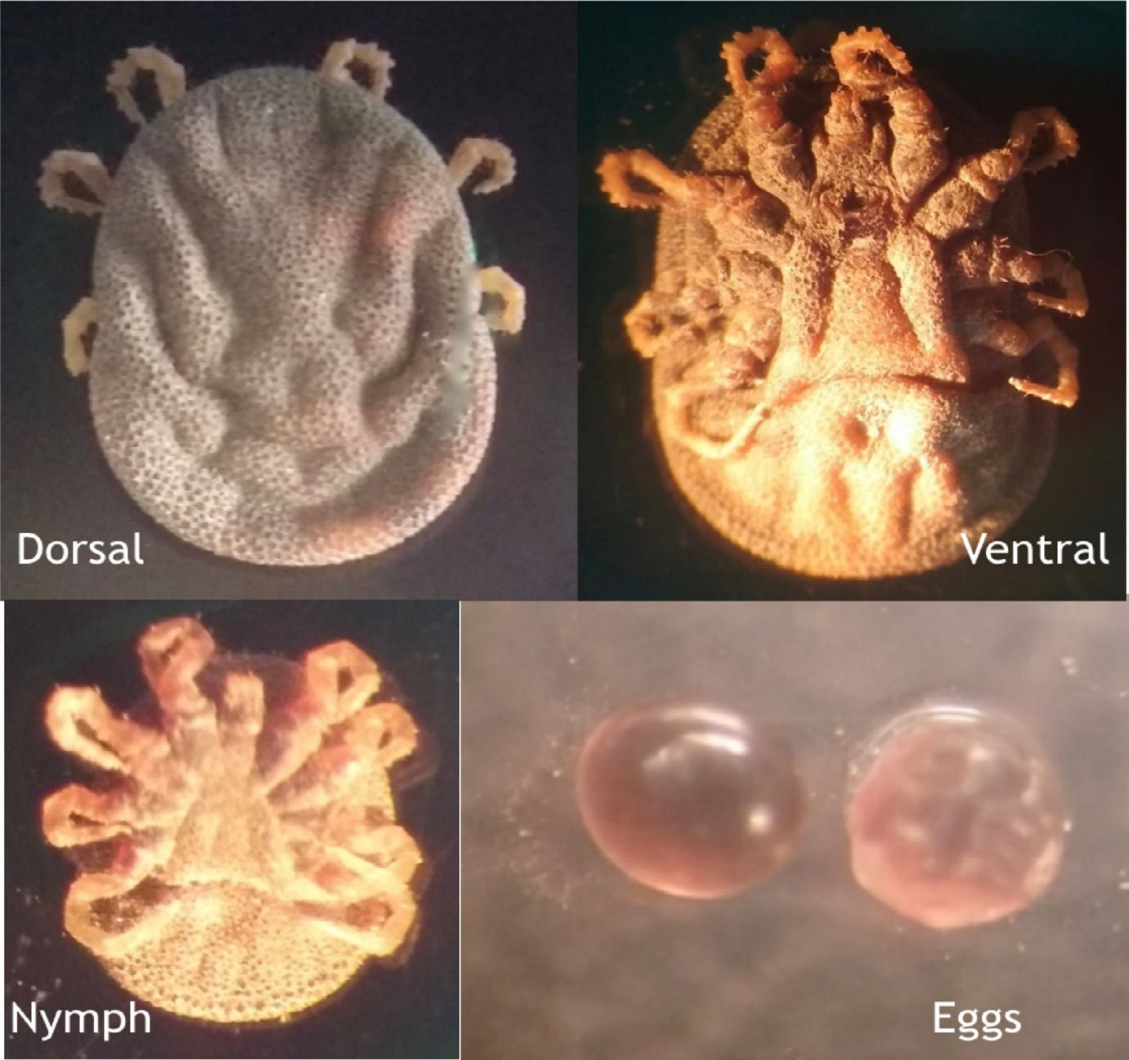
Images showing features that are typical for ticks of the *Ornithodoros* complex group (taken under 20× magnification of a compound microscope)

Upon collection of tick samples from warthog burrows, all ticks from one burrow were placed in one tube. Morphological identification of the ticks was performed using a stereomicroscope (Wild, Heerbrugg, Switzerland) by analysing the characteristic features associated with the *Moubata* complex group. The observed features which are typical characteristics of the *O. moubata* complex group included leathery and wrinkled dorsal surface covered with nodules, lack of eyes, absence of a rigid scutum and mouth not visible from above (Figure 2).

Total genomic DNA was extracted from intact ticks preserved in 70% ethanol using DNeasy Blood and Tissue Kit^®^ (Qiagen) with some modification on the preparation of the ticks before extraction. Ticks were removed from 70% ethanol, dried on a blotting paper and placed in a clean 50 ml falcon tube. The tube was topped with 25 ml Milli‐Q^®^ water and shaken slowly to avoid breakage of the ticks. Washing was repeated three times. Washed ticks were spread on a blotting paper and left to air‐dry for 15 min. Cleaned and dried ticks were put in 1.5 ml Eppendorf tubes. To obtain DNA of sufficient quality and quantity, only large ticks of 5‐8 mm width were analysed in this study. Using an empty Eppendorf tube, a small amount of liquid nitrogen was added into the tube containing a tick and ground using a sterile plastic pestle (Bel‐Art products, New Jersey, USA) into a fine powder. To the tick powder, 180 μl Buffer ATL was added, followed by 20 μl Proteinase K enzyme. Samples were incubated in a shaking water bath at 56°C overnight to allow complete digestion of the samples. The DNA samples were aliquoted and stored at −20ºC.

ASFV DNA was confirmed by PCR amplification of a 257bp region corresponding to the C‐terminal region of the major capsid protein encoded by B646L gene using the diagnostic primers PPA1 and PPA2 as previously described (Agüero et al., 2003). PCR reactions were performed in a total volume of 25 μl.

#### 2.5.4 Genotyping and sequence analysis

To characterize the ASF virus, samples that were PCR‐positive for ASFV using diagnostic primers PPA1 and PPA2 were subjected to the ASFV genotyping PCR that targeted three polymorphic loci. The first locus is the C‐terminal region of B646L gene that encodes the major capsid protein p72 by using primers p72‐U (5’GGCACAAGTTCGGACATGT3’) and p72‐D (5’GTACTGTAACGCAGCACAG3’) which amplify a 478bp DNA fragment (Bastos et al., 2003). The second locus is the complete E183L open reading frame that encodes the p54 ASFV protein, which is used to place the ASFV in major subgroups. The gene was amplified using the primers PPA722 (5’CGAAGTGCATGTAATAAACGTC3’) and PPA89 (5’TGTAATTTCATT GCGCCACAAC3’) flanking a 676bp DNA fragment (Gallardo et al., 2009). Lastly, further genotypic discrimination was done by amplification of the B602L gene for the central variable region (CVR). Analysis of this region was done by translation of the amplicon sequences into amino acids. Tetrameric amino acid repeats known to exist in the CVR of ASFV isolates include repeat codes CAST/CVST/CTST (A), CADT/CTDT (B), GAST/GANT (C), CASM (D), CANT (F), CTNT (G), NEDT (M), NVDT/NVGT/ NVNT (N), NANI/NADI/NASI (O), RAST (H), SAST (S), NVNT (T), NAST/NADT/NANT (V) SADT/ SVDT (W) NIDT/NTDT (U) and NTDI (X) (Lubisi et al., 2007; Misinzo et al., 2011).

Amplicons of the expected size were purified using QIAquick PCR purification Kit (Qiagen, Hilden, Germany) following the manufacturer's instructions and sent to Bioneer Corporation for Sanger sequencing. A Basic Local Alignment Search Tool (BLAST) was used to search sequence similarity with other ASFV in the GenBank database. Similar sequences were downloaded with their respective accession numbers and used for comparison. For improved specificity, only Tanzanian isolates were included in the analysis. Multiple sequences alignment was generated with sequences from this study, and Tanzanian ASFV reference sequences retrieved from the GenBank (Lubisi et al., 2007; Misinzo et al., 2011) using the ClustalW alignment tool (Thompson, Higgins, & Gibson, 1994). Three phylogenetic data sets were generated for the p72 gene (B646L), central variable region (B602L) and p54 gene (E183L). Sequences from the ASFV genotype XV used were the 2008 outbreak isolates from Mazimbu, Mabibo and Turiani with the exception of B602L Turiani isolate that was not available in the database. Construction of phylogenetic trees was done by the neighbour‐joining method using Kimura‐2‐parameter option generated within MEGA 6 software (Tamura, Stecher, Peterson, Filipski, & Kumar, 2013).

## 3 RESULTS

### 3.1 Analysis of warthog sera for ASFV antibody by indirect ELISA

**FIGURE 3:**
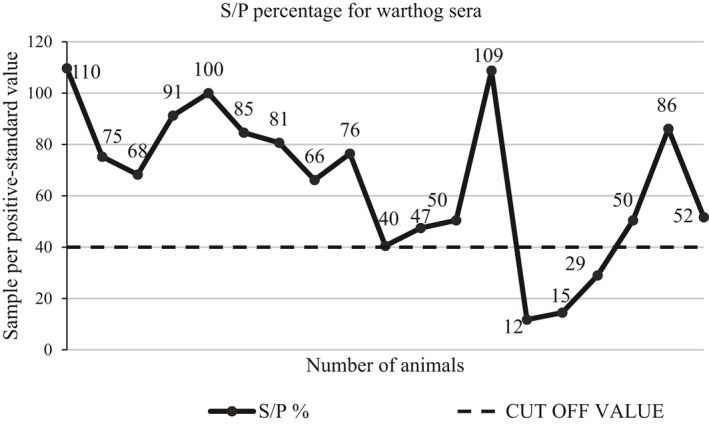
Seroprevalence of ASFV antibody in warthog sera. Sixteen of the 19 warthog sera tested by ELISA were positive for antibodies against ASFV, corresponding to a seroprevalence of 84%. S/P%, sample per positive standard percentage

Warthog sera samples were analysed for the presence of antibodies against ASFV by indirect ELISA. Sixteen out of 19 (84%) warthog sera were found positive for antibodies against ASFV, with sample per positive standard percentage (S/P%) values of 40% and above (Figure 3). The distribution of sex and age groups of animals sampled is shown in Table 1. Of the 16 positive samples, 14 (87.5%) had an S/P% higher than 50, and two samples had an S/P% of above 100%, as shown in Figure 3, interpreted as intense exposure of warthogs to the virus. However, when blood samples from the same animals were tested by PCR, none of the 19 samples was positive for the presence of the viral DNA.

**TABLE 1:** A summary of ELISA results for the 19 warthogs sampled in Saadani National Park

Sex	ASFV antibody‐ELISA				Total
	Adult warthogs		Juvenile warthogs (<6 months)		
	Positive	Negative	Positive	Negative	
Male	2	1	2	0	5
Female	9	0	3	2	14
Total	11	1	5	2	19

Abbreviations: ASFV, African swine fever virus.

### 3.2 Identification and classification of ticks

Five active warthog burrows were sampled, and 300 ticks were collected, of which 111 ticks were large enough to be analysed. The number of ticks recovered from a burrow ranged from 12 to 48 (Table 2). From the microscopic examination of morphological characteristics, it was possible to classify ticks to within the *O. moubata* complex group, but not possible to identify down to the genus level (Figure 2). A total of 111 large ticks were characterized individually.

**TABLE 2:** Descriptions of ticks collected from warthog burrows indicating their feeding status and the overall ASFV prevalence using PCR

Burrow	Ticks		PCR positive	
	Total	Engorged	Engorged ticks	Total ASFV positive (prevalence)
1	48	31	5	9 (18.7%)
2	20	15	2	2 (10.0%)
3	17	4	0	3 (17.6%)
4	14	10	3	3 (21.4%)
5	12	9	3	3 (25.0%)
Total	111	69	13	20 (18%)

### 3.3 | Diagnostic PCR for ASFV in tick samples

One positive tick sample per burrow was sequenced for the p72, p54 and CVR gene regions. One independent tick from each of the five selected samples had a low‐quality sequence in two of the three sequenced regions and hence was excluded from the analysis.

Of the 111 ticks, 62% (69) were engorged having had a blood meal before their collection (Table 2). A total of 20 out of 111 (18%) ticks individually analysed were found positive for ASFV by conventional PCR, of which seven (35% of all positive) ticks were not engorged.

### 3.4 Genotyping of viral DNA from ticks

#### 3.4.1 Sequencing of the p72 (B646L) gene

**FIGURE 4:**
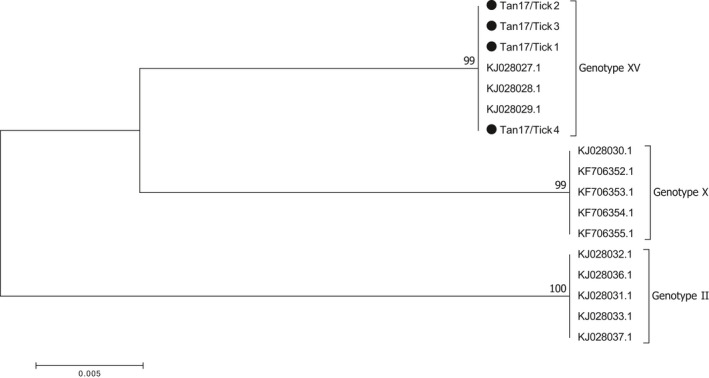
P72 (B646L) phylogenetic tree showing the relationship between sequences from this study (•) with reference sequences from previous Tanzania ASF outbreaks (including sequences from 2008 that clustered under genotype XV). The phylogeny was inferred following 1,000 bootstrap replications, and the node values show high percentage bootstrap support. The scale bar indicates nucleotide substitutions per site

Phylogenetic analysis of the p72 region of the virus showed clustering of the Saadani sequences obtained in this study with sequences from the 2008 ASF outbreaks in Tanzania (Figure 4), with strong support for the sequences being of genotype XV origin (Misinzo et al., 2011).

#### 3.4.2 Sequencing of the p54 (E183L) gene

**FIGURE 5:**
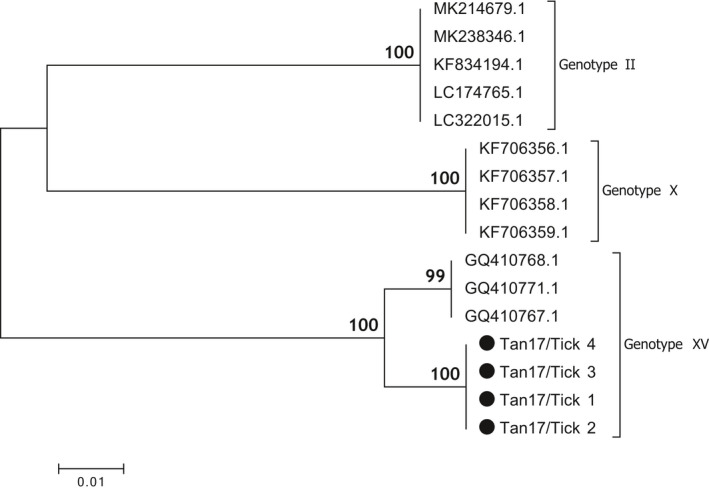
Phylogenetic tree of p54 (E183L) DNA sequences from Ticks (•) and reference sequences for genotypes II, X and XV, retrieved from GenBank. The phylogeny was inferred following 1,000 bootstrap replications, and the node values show high percentage bootstrap support. The scale bar indicates nucleotide substitutions per site

Sequence analysis of E183L gene (encoding the antigen p54) was used to differentiate between closely related ASF viruses of the same genotype due to the highly conservative nature of this region. Sequences obtained from this study clustered under the same genotype XV as those from the 2008 ASF outbreaks in Tanzania at a bootstrap value of 100% (Figure 5).

#### 3.4.3 Sequencing of the Central Variable Region (CVR) B602L gene

**FIGURE 6:**
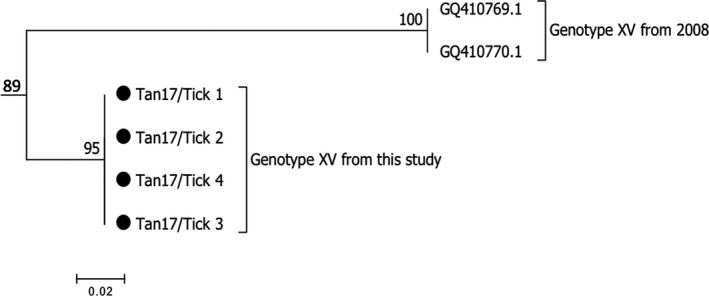
Bootstrap relationship for the B602L CVR region for genotype XV sequences from Ticks (•) and the 2008 Tanzania outbreak references from GenBank. The scale bar indicates nucleotide substitutions per site

Phylogenetic analysis of the CVR was done by comparing the study sequences with sequences from the 2008 outbreak. CVR sequences from this study clustered with the references at a bootstrap value of 89% but subclustered into two distinct clusters, as shown in Figure 6. The result of an in‐depth analysis of the predicted amino acids from the region by counting the tetrameric amino acid repeats known to exist in the CVR of ASFV isolates is shown in Table 3.

**TABLE 3:** Tetrameric repeats of amino acid sequences obtained from the CVR of a genotype XV ASFV collected from Saadani (shorter) and from 2008 outbreak (longer)

ASFV Isolate	Tetrameric repeat	Number of repeats
Tan17/Tick 1	AVUAVUVVAVVVVAVVUV	18
Tan17/Tick 2	AVUAVUVVAVVVVAVVUV	18
Tan17/Tick 3	AVUAVUVVAVVVVAVVUV	18
Tan17/Tick 4	AVUAVUVVAVVVVAVVUV	18
MAZIMBU	AVUAVUVAVVUAVUVAVUVAVVUAVVUUUXV	31
MABIBO	AVUAVUVAVVUAVUVAVUVAVVUAVVUUUXV	31

## 4 DISCUSSION

The findings of this research contribute to our understanding of African swine fever in Tanzania, which up until now has focused primarily on interactions between sylvatic hosts and domestic pigs (Bastos et al., 2009; Jori & Bastos, 2009; Plowright et al., 1969). This study detected ASFV infections in ticks (18%) along with a high ASFV seroprevalence in warthogs (84%), including juvenile animals, indicating that ASFV is circulating within a tick‐warthog cycle in Tanzania in the absence of potential transmission to and from domestic pigs. High seroprevalence in warthogs has also been observed in Serengeti National Park (Katale et al., 2012). Our results indicate that ASF is likely maintained independently within sylvatic hosts and that probably, the sylvatic cycle plays an essential role in the maintenance of the ASF virus, consistent with findings from other parts of Eastern Africa (Anderson et al., 1998; Bastos et al., 2009; Okoth et al., 2012; Plowright et al., 1969). Similar conclusions have been drawn from studies in southern Africa region including Malawi, Zambia and Mozambique (Anderson et al., 1998; Boshoff, Bastos, Dube, & Heath, 2014; Gallardo et al., 2011; Haresnape, Wilkinson, & Mellor, 1988; Quembo et al., 2018; Stahl et al., 2014).

The finding of ASFV infection in a tick‐warthog cycle would raise concerns about the potential for spill‐over to domestic pigs if these were to be introduced into the area. This finding has important implications for community outreach programmes involving diversification of livelihoods through the introduction of pig farming. In another study, it was suggested that domestic pigs adjacent to protected areas in Northern Tanzania were at a higher risk of contracting the disease (Katale et al., 2012). While the small sample size limits the precision and representability of the results, the finding of infection in ticks and warthogs, even at a small scale, provides evidence of the existence of an influential sylvatic maintenance community across broader scales although its distribution within the park boundaries could not be assessed.

The detection of antibodies but not ASFV nucleic acid in warthog blood is consistent with other studies which show that warthogs can clear ASFV (or maintain the virus at a level undetectable by conventional PCR) in their blood (Jori & Bastos, 2009; Thomson, 1985), Figure 6.

Earlier studies have shown that a high viraemia was achieved in warthog piglets that spend more time in the burrows during early stages of life (Thomson, Gainaru, & Dellen, 1980). Unfortunately, the timing of this study did not allow for the capture of young piglets under six months of age. While none of the juveniles was viraemic in this study, further work is needed to clarify the role of transmission in young piglets in this system.

The ASF virus is known to be maintained in the ticks transovarially and transstadially (Kleiboeker & Scoles, 2001), which explains our observation that seven out of 20 non‐engorged PCR‐positive ticks were also positive for ASFV (Table 2). Even though this prevalence is derived from the small sample of burrows, the high levels of exposure in warthogs (84%) may explain the efficiency of this biological system in maintaining and transmitting the virus even with a low prevalence (18%) of infected ticks.

The current study did not show a clear association between sex and age of warthogs to ASFV exposure status. This can partly be due to the small sample size that was not representative enough for different age groups and sex (Table 1) and also probably because none of the animals in any category was found in active viraemia state.

Genotype XV described in the current study has been reported in previous studies in Tanzania (Misinzo et al., 2011, 2014; Yona, 2017). The p72 references from GenBank and the sequences from this study were highly similar, clustering together under genotype XV with the bootstrap value of 99% (Figure 4). Upon a BLAST search of the tick‐derived ASFV sequences, there was 100% identity with the Tanzanian genotype XV references. The BLAST search of the p54 region that is much more conserved at the strain level returned a 95% identity with other genotype XV sequences from Tanzania and the sequences clustered under different branches of the same subtree (Figure 5). An analysis of the CVR showed variations in both numbers of the tetrameric amino acid repeats and their sequences. Despite strong bootstrap support of 89% that the sequences belonged in the same group, a phylogenetic tree showed a difference in sub‐clustering of the sequences from this study with those from the 2008 outbreak (Figure 6). The first seven tetrameric repeats were identical in both the references and the sequences from this study showing a level of relatedness within the sequences. Sequences from this study appeared to be shorter, with 18 tetrameric repeats as compared to the references that had 31 repeats (Table 3). The results could not conclude whether such variation would result in a more or less virulent strain of the virus.

Based on the findings emanating from the close relationship between sequences from this study, several hypotheses can be formulated. The virus which caused an outbreak in pigs in 2008 could have originated from another sylvatic source different from the one in Saadani National Park that is yet to be identified. We consider it unlikely that the source of this outbreak originated in Saadani because there are not any ongoing pig rearing activities that would enhance transmission in this area.

Another hypothesis is that the virus in the sylvatic cycle may have spilt over into the domestic pigs, perhaps through illegal hunting of warthogs and distribution of warthog meat, causing an ASF outbreak in domestic pigs. The sylvatic cycle in Saadani could represent a long‐standing uninterrupted cycle that has maintained itself for many years independent of any other interactions.

Our results implicate genotype XV as potentially infective to warthogs as evidenced by seropositivity in warthog sera (Figure 3). Although the current study cannot make any inference about the virulence of this specific sylvatic strain in domestic pigs, this remains to be experimentally determined. Further research on the directional flow of ASFV between the sylvatic and domestic cycles, its virulence and the role that wild hosts and vectors play in modifying the virus needs to be further undertaken.

Despite the small sample size, this study provides valuable insights into the sylvatic cycle in closed virus transmission systems. Further work is needed to compare whole virus genomes of genotype XV from ticks in Saadani National Park with entire genome sequences of isolates from 2008 outbreaks to provide more insights into the ASF virus evolution and transmission dynamics. Studies on the virulence of genotype XV are needed to assess its potential for local and global spread and risks. Diversification of livelihoods through the introduction of pig farming in the study area will require community outreach programmes addressing ASF control in order to avoid the risk of the disease.

## CONFLICT OF INTEREST

The authors declare that there is no conflict of interest that could be perceived as prejudicing the impartiality of the research reported.

## ETHICAL STATEMENT

Relevant permits to conduct the study in Saadani National Park were obtained from Tanzania Wildlife Research Institution (TAWIRI) and Tanzania National Parks Authority (TANAPA).

## Data Availability

The data that support the findings of this study are openly available in NCBI at https://www.ncbi.nlm.nih.gov/genbank, GenBank accessions MT396736‐MT396747 under the GenBank BankIt submission ID: 2168824.
